# Cytotoxic corpus callosum lesion and mild CSF pleocytosis during hantavirus infection: a case report

**DOI:** 10.1177/17562864221144808

**Published:** 2022-12-26

**Authors:** Frederike A. Straeten, Gerd Meyer zu Hörste

**Affiliations:** Department of Neurology with Institute of Translational Neurology, University Hospital Muenster, 48149 Muenster, Germany; Department of Neurology with Institute of Translational Neurology, University Hospital Muenster, Muenster, Germany

**Keywords:** case report, clinical neurology, cytotoxic corpus callosum lesion, hantavirus, MRI

## Abstract

A middle-aged, previously healthy male patient presented with high fever, headache, and aching limbs for 3 days. Laboratory results showed signs of acute kidney injury, elevated procalcitonin, and mild thrombocytopenia. On neurological examination, he had no focal neurological deficits, especially no meningism or visual disturbances. Cerebrospinal fluid (CSF) examination showed mild lymphocytic pleocytosis, and magnetic resonance imaging (MRI) revealed a lesion of the splenium corporis callosum. The patient received anti-infective treatment with acyclovir and ceftriaxone until laboratory results returned positive hantavirus IgM and IgG antibodies in the serum indicating an active hantavirus infection. The renal retention parameters and thrombocytopenia receded following treatment with intravenous fluids, analgesic, and antipyretic agents. MRI follow-up 10 days later showed a residual small FLAIR-positive lesion without any persistent callosal diffusion abnormality. The patient was discharged symptom-free after 8 days and had recovered fully 2 months later. The source of infection in this patient remained unclear. Cytotoxic lesions of the corpus callosum (CLCC) are secondary lesions usually with a good prognosis but require further investigation regarding their underlying etiology and should not be confounded with primary callosal lesions, such as ischemia or lymphoma.

*We used the CARE checklist when writing our report.* ^1^

## Introduction

Hantavirus are enveloped RNA viruses and comprise 28 known human pathogen strains worldwide. In humans, hantavirus infections cause hemorrhagic fever with renal syndrome and hantavirus cardiopulmonary syndrome. They both ground in the pathogenesis of increased vascular permeability due to deregulation of endothelial barrier functions and thrombocytopenia as a result of platelet dysfunction.^2^ Puumala hantavirus (PUUV) in specific causes a mild variant of hemorrhagic fever with renal syndrome. This strain is endemic in Europe and transmitted via inhalation of aerosols from excretions or saliva of its rodent host, the bank vole.^3^ The infection manifests with acute onset of high fever, acute kidney injury, and thrombocytopenia.^2^ Acute organ injury during PUUV is mediated by cytokines, platelet dysfunction, and the dysregulation of endothelial cell barrier.^2^ Accompanying neurological complications, such as abnormalities of cerebral imaging or cerebrospinal fluid (CSF), is scarcely reported and not well-defined yet. We here describe a cytotoxic lesion of the corpus callosum with mild lymphocytic CSF pleocytosis during hantavirus infection.

### Patient information

The middle-aged patient with no previous medical history presented to the emergency department of a tertiary care hospital with intense headache, high fever up to 40.6°C (105.1°F), and aching limbs for 3 days. The headache was described as throbbing (maximum on numeric rating scale 9/10) without concomitant photo-, phonophobia, or vomiting. A reduction of vigilance or signs of meningeal affection (Laségue-, Brudzinski- or Kernig-sign) were not observed. The body and neurological examination were otherwise unremarkable. The patient denied closer contact to people suffering of any infection or any animals, and his travel and drug history were unremarkable. He had not done recent gardening work.

### Diagnostic assessment

The course of laboratory parameters is shown in Table 1. The patient had extensive proteinuria [urine albumin/creatinine ratio 1496.2 (<30)].

**Table 1.** Laboratory chemical evaluation during the hospital stay.

Blood sampling	Day of admission	Day 3	d6	At discharge
Leukocytes (3.91–10.9 Tsd/µl)	7.74	10.94	8.59	6.53
Hemoglobin (13.5–16.9 g/dl)	15.9	12.1	11.7	12.1
Hematocrit (40–49, 4%)	45.2	33.3	33.8	35.7
**Platelets (166–308 Tsd/µl)**	**102**	**124**	**325**	**434**
**Creatinine (<1.3 mg/dl)**	**1.3**	**2.4**	**1.6**	**1.3**
Bilirubin, total (<1.2 mg/dl)	1	NA	0.4	0.4
GOT (AST) (10–50 U/liter)	60	326	40	31
Gamma-GT (<66 U/liter)	60	123	110	96
Alkaline phosphatase (40–129 U/liter)	77	NA	NA	NA
CK (<174 U/liter)	110	NA	NA	NA
**eGFR (CKD-EPI) (>90)**	**70**	**33**	**55**	**70**
**CRP (<0.5 mg/dl)**	**12.6**	**2.8**	**5**	**1.9**
Haptoglobin (30–200 mg/dl)	NA	NA	203	NA
TSH (0.27–4.2 µU/ml)	1.53	NA	NA	NA
**Procalcitonin (<0.5 ng/ml)**	**1.27**	NA	**0.14**	NA

NA, not acquired, AST, aspartate aminotransferase, CDK-EPI, equation to estimate the GFR, CK, creatine kinase, eGFR, estimated glomerular filtration rate, GOT, glutamic oxaloacetic transaminase, TSH, thyroid-stimulating hormone.

After initial thrombocytopenia, cell levels rose above the upper limit of normal. Renal parameters continued to drop after admission before they began to recover again. The bold values reflect relevant laboratory abnormalities during the infection.

**Figure 1.**
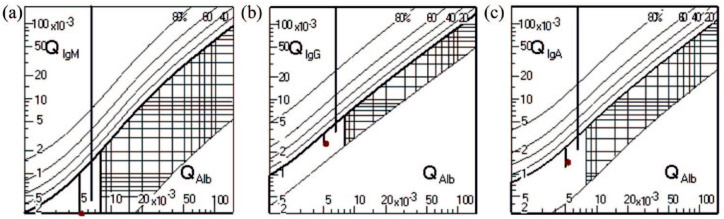
Projection of the immunoglobulin (Ig) CSF/Ig serum index compared to the albumin CSF/albumin serum index at the time of sample collection. The sustained selectivity of the blood–brain barrier is derived via controlling the CSF albumin/CSF serum index as albumin is solely produced peripherally and therefore the CSF concentration is sensitive to changes in the CSF barrier function (CSF flow). The curve is depicted for each Ig separately. (a) Showing the Reiber scheme for IgM, (b) for IgG, and (c) for IgA. The red dots indicate the calculated quotient. All values indicate an intact blood–brain barrier.

The CSF contained 11 cells/µl (<5/µl) with 9 lymphocytes/µl, 1 granulocyte/µl, and 1 other cell/µl. Total protein was 386 mg/liter (200–500 mg/liter) (see Table 2). On Reiber scheme, which is projecting the immunoglobulin (Ig) CSF/Ig serum index compared to the albumin CSF/albumin serum, there was no hint of blood–brain barrier disruption (see Figure 1). PCR (polymerase chain reaction) investigations for common viral or pathogens [cytomegalovirus (CMV), Epstein–Barr virus (EBV), herpes simplex virus 1 and 2 (HSV-1, -2), varicella-zoster virus (VZV), human herpesvirus 6 (HHV-6)] in the CSF were negative. Antigen production against tick-borne encephalitis (TBE), *Borrelia burgdorferi*, *Treponema pallidum*, Leptospira, Legionella, or Pneumococci was not detectable.

**Table 2.** CSF sampling showing mild lymphocytic pleocytosis.

CSF parameters	On the day of admission
Cell count (<5/µl)	11/µl
Lymphocytes	9/µl
Granulocytes	1/µl
Erythrocytes	35/µl
Other cells	1/µl
Total protein (200–500 mg/liter)	386 mg/liter
Albumin CSF	204 mg/liter
Albumin serum	38.6 g/liter
Albumin quotient	5.3
IgG CSF	27.2 mg/liter
IgG serum	10.5 g/liter
IgG quotient	2.6
IgA CSF	2.89 mg/liter
IgA serum	2.02 g/liter
IgA quotient	1.4
IgM CSF	0.24 mg/liter
IgM serum	1.05 g/liter
IgM quotient	0.2
OCB	Type 1
Glucose CSF (49–75 mg/dl)	74.8
Glucose serum (70–130 mg/dl)	117
Glucose quotient (0.6–0.9)	0.64
Lactate (1.5–2.1 mmol/liter)	1.89
Bilirubin	Negative

CSF, cerebrospinal fluid; OCB, oligoclonal bands.

Total cell count was slightly elevated and predominantly consisted of lymphocytes.

**Figure 2.**
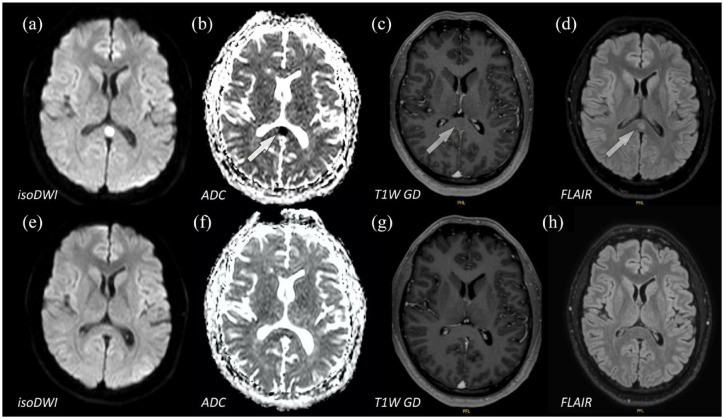
(a–d) Showing brain MRI on day 2, (e and f) follow-up MRI on day 13. On day 2, there was a signal intense lesion of the splenium corporis callosi on (a) isotropic diffusion-weighted sequences, (b) with correlating ADC reduction. (c, g) Contrast medium enhancement was not present. (d) Correlating findings were seen on FLAIR sequences with an ovoid symmetric lesion of the callosum. (h) After 11 days, only slight residual signal enhancement on FLAIR sequences due to the radiological report was present. ADC, apparent diffusion coefficient; FLAIR, fluid-attenuated inversion recovery; isoDWI, isotropic diffusion-weighted imaging; T1W GD, T1-weighted gadolinium.

MRI showed a single lesion of the corpus callosum with apparent diffusion coefficient (ADC) reduction and correlating signal enhancement on isotropic diffusion-weighted sequences. After 10 days, this lesion had significantly receded. Only slight residual fluid-attenuated inversion recovery (FLAIR) hyperintense signs were detectable (see Figure 2).

### Differential diagnosis

Reported infectious causes of cytotoxic lesions of the corpus callosum (CLCC) have been described secondary to other infectious agents, including adenovirus, EBV, *Escherichia coli*, herpes infections, influenza virus A (H1 N1), legionella, malaria, measles, mycoplasma, mumps, rotavirus, salmonella, *Staphylococcus*, *Streptococcus*, TBE, and VZV.^4^ We conducted extensive virus diagnostics and bacteriological examination without a further pathogen detection. Rheumatological screening was negative. Due to the absence of a positive travel history, malaria was improbable.

Reversible splenial lesion syndrome (RESLES) has been described in 2011.^5^ It was classified as clinicoradiological syndrome due to antiepileptic drug (AED) withdrawal, infection, high-altitude cerebral edema, or metabolic disorder – hypoglycemia and hypo- or hypernatremia in specific. RESLES in association with infection was clinically related with encephalopathy or encephalitic features. The predominant causative agent – if determined – was viral, particularly influenza virus.^5^ Mild encephalopathy with reversible splenial lesion (MERS) is considered a subtype of RESLES sharing the same etiologies – particularly of viral origin – but with the additional diagnostic criteria of a consisting mild encephalopathy or encephalitis.^6,7^ This nomenclature may confuse during clinical practice for several reasons. First, the lesions may extend beyond the splenium into the white matter.^8^ Second, CLCC are not always reversible and may result in gliosis.^9^ Finally, the encephalopathy may range from being absent to severe.^4^

### Therapeutic intervention

Because of mild lymphocytic pleocytosis, the patient received anti-infective treatment with intravenous ceftriaxone, acyclovir, and ampicillin. After serological detection of positive IgM and IgG hantavirus antibodies (serotype Puumala) via western blot, anti-infective therapy was discontinued.

### Follow-up and outcome

After 8 days, the patient was discharged. Clinically, the patient had recovered fully without any persisting symptoms or signs. However, 2 months later, the patient was still free of symptoms and physically fit. The source of infection in this patient remained unresolved.

## Discussion

The clinical manifestation of PUUV commonly includes fever, headache, gastrointestinal symptoms, and impaired renal function.^3^ To our knowledge, there are two published case reports – from 2012 and 2019 – about CLCCs caused by PUUV infection. In both cases, no lumbar puncture was performed.^10,11^

CLCCs are secondary lesions usually with a good prognosis associated with drugs, malignancies, metabolic disorders, subarachnoid hemorrhage, or infections.^4,5^ Radiologically, they are characterized as areas of low diffusion on diffusion-weighted MRIs due to cytotoxic edema. This edema is explained by a cytokinopathy resulting in a high rise in extracellular glutamate levels (up to 100 times above the upper normal limit).^4^ Analysis of the cytokine profile revealed a pronounced T_H_17-(IL-6), T_H_1-(CXCL10, TNF-α, IFN-γ) and neutrophil-activation (IL-8, CXCL1) in the CSF and a T_H_1-(CXCL10, TNF-α, IFN-γ), T_H_17-(IL-6) and inflammasome-response (IL-1β) in the serum.^12^ In these analyzed patients, hypercytokinemia was accompanied by hyponatremia, potentially contributing to a lower threshold of edema development in addition.^8,12^ CLCCs do not show contrast material enhancement and are relatively symmetric.^4,13^ The first published and initially most frequently observed cause of callosal edema were AEDs, especially the reduction of AED dose.^8,13^ Currently, they are not regarded as specific for one clinical condition, nevertheless most likely sharing the common pathophysiologic mechanism of cytotoxic edema induced by cytokine and immune cell activation.^13^

To conclude, CLCC are secondary lesions usually with a good prognosis associated with drugs, malignancies, metabolic disorders, subarachnoid hemorrhage, or infections. They always require further investigation regarding their underlying etiology and should not be confounded with primary callosal lesions, such as ischemia or lymphoma. The prevalence of cerebral and CSF abnormalities during PUUV infection is actually unknown but might me more common than previously anticipated.

### Multiple choice question

Secondary CLCC should not be confounded with primary lesions, such as ischemia. Which are the imaging characteristics aiding the differentiation of the latter from CLCCs?

1. CLCCs are always completely reversible, whereas ischemic lesions are not.
2. CLCCs tend to be midline and are relatively symmetric, whereas ischemic lesions often show a paramedian and more lateralized location.
3. The correlate of an ischemic FLAIR-hyperintense lesion is the area of low diffusion (ADC reduction). CLCCs are distinct from that as they show no area of reduced diffusion.
4. CLCCs are characterized by their contrast medium enhancement which is not observed by ischemic lesions.

Answer:

1. *CLCCs are not always completely reversible.* ^4^
2. *Correct answer.* ^4,14^
3. *CLCCs and ischemic lesions exhibit an area of low diffusion/ADC reduction.* ^4,14^
4. *CLCCs do not show contrast material enhancement, whereas intravascular enhancement on contrast-enhanced T1-weighted sequences can be observed in the acute phase of stroke due to decelerated arterial blood flow.* ^4,15^

## Supplemental Material

sj-docx-1-tan-10.1177_17562864221144808 – Supplemental material for Cytotoxic corpus callosum lesion and mild CSF pleocytosis during hantavirus infection: a case report Click here for additional data file. Supplemental material, sj-docx-1-tan-10.1177_17562864221144808 for Cytotoxic corpus callosum lesion and mild CSF pleocytosis during hantavirus infection: a case report by Frederike A. Straeten and Gerd Meyer zu Hörste in Therapeutic Advances in Neurological Disorders

## Declarations

**Ethics approval and consent to participate:** Not applicable; no ethics committee approval was required for this report, as it was a retrospective description of a single case.

**Consent to publication:** The patient gave written informed consent to publish this case report.

**Availability of data and materials:** The authors have full access to the patient data.
