# Supplementary material for: Cytotoxic corpus callosum lesion and mild CSF pleocytosis during hantavirus infection: a case report
Source: Ther Adv Neurol Disord. 2022 Dec 26;15:17562864221144808. doi: 10.1177/17562864221144808 (PMC9806393; doi:10.1177/17562864221144808)
Supplement: sj-docx-1-tan-10.1177_17562864221144808 – Supplemental material for Cytotoxic corpus callosum lesion and mild CSF pleocytosis during hantavirus infection: a case report [file sj-docx-1-tan-10.1177_17562864221144808.docx]

# Reporting checklist for case report or case series.

Based on the CARE guidelines.

## Instructions to authors

Complete this checklist by entering the page numbers from your manuscript where readers will find each of the items listed below.

Your article may not currently address all the items on the checklist. Please modify your text to include the missing information. If you are certain that an item does not apply, please write "n/a" and provide a short explanation.

Upload your completed checklist as an extra file when you submit to a journal.

In your methods section, say that you used the CAREreporting guidelines, and cite them as:

Gagnier JJ, Kienle G, Altman DG, Moher D, Sox H, Riley D; the CARE Group. The CARE Guidelines: Consensus-based Clinical Case Reporting Guideline Development

|  |  | Reporting Item | Page Number |
| --- | --- | --- | --- |
| **Title** |  |  |  |
|  | [#1](https://www.goodreports.org/reporting-checklists/care/info/#1) | The area of focus and “case report” should appear in the title | 1 |
| **Keywords** |  |  |  |
|  | [#2](https://www.goodreports.org/reporting-checklists/care/info/#2) | Two to five key words that identify topics in this case report | 1 |
| **Abstract** |  |  |  |
| Introduction | [#3a](https://www.goodreports.org/reporting-checklists/care/info/#3a) | What is unique and why is it important? | 1 |
|  | [#3b](https://www.goodreports.org/reporting-checklists/care/info/#3b) | The patient’s main concerns and important clinical findings. | 1 |
|  | [#3c](https://www.goodreports.org/reporting-checklists/care/info/#3c) | The main diagnoses, interventions, and outcomes. | 1 |
| Conclusion | [#3d](https://www.goodreports.org/reporting-checklists/care/info/#3d) | What are one or more “take-away” lessons? | 1 |
| **Introduction** |  |  |  |
|  | [#4](https://www.goodreports.org/reporting-checklists/care/info/#4) | Briefly summarize why this case is unique with medical literature references. | 1 |
| **Patient information** |  |  |  |
|  | [#5a](https://www.goodreports.org/reporting-checklists/care/info/#5a) | De-identified demographic and other patient information. | 1, 2 |
|  | [#5b](https://www.goodreports.org/reporting-checklists/care/info/#5b) | Main concerns and symptoms of the patient. | 1, 2 |
|  | [#5c](https://www.goodreports.org/reporting-checklists/care/info/#5c) | Medical, family, and psychosocial history including genetic information. | 1, 2 |
|  | [#5d](https://www.goodreports.org/reporting-checklists/care/info/#5d) | Relevant past interventions and their outcomes. | 1, 2 |
| **Clinical findings** |  |  |  |
|  | [#6](https://www.goodreports.org/reporting-checklists/care/info/#6) | Relevant physical examination (PE) and other clinical findings. | 1, 2 |
| **Timeline** |  |  |  |
|  | [#7](https://www.goodreports.org/reporting-checklists/care/info/#7) | Relevant data from this episode of care organized as a timeline (figure or table). | 2, 3 |
| **Diagnostic assessment** |  |  |  |
|  | [#8a](https://www.goodreports.org/reporting-checklists/care/info/#8a) | Diagnostic methods (PE, laboratory testing, imaging, surveys). | 2, 3 |
|  | [#8b](https://www.goodreports.org/reporting-checklists/care/info/#8b) | Diagnostic challenges. | 4 |
|  | [#8c](https://www.goodreports.org/reporting-checklists/care/info/#8c) | Diagnostic reasoning including differential diagnosis | 4 |
|  | [#8d](https://www.goodreports.org/reporting-checklists/care/info/#8d) | Prognostic characteristics when applicable | 4 |
| **Therapeutic Intervention** |  |  |  |
|  | [#9a](https://www.goodreports.org/reporting-checklists/care/info/#9a) | Types of intervention (pharmacologic, surgical, preventive). | 4 |
|  | [#9b](https://www.goodreports.org/reporting-checklists/care/info/#9b) | Administration of intervention (dosage, strength, duration) | N/A |
|  | [#9c](https://www.goodreports.org/reporting-checklists/care/info/#9c) | Changes in the interventions with explanations. | 4 |
| **Follow up and outcomes** |  |  |  |
|  | [#10a](https://www.goodreports.org/reporting-checklists/care/info/#10a) | Clinician and patient-assessed outcomes when appropriate | 4 |
|  | [#10b](https://www.goodreports.org/reporting-checklists/care/info/#10b) | Important follow-up diagnostic and other test results. | 4 |
|  | [#10c](https://www.goodreports.org/reporting-checklists/care/info/#10c) | Intervention adherence and tolerability (how was this assessed)? | N/A |
|  | [#10d](https://www.goodreports.org/reporting-checklists/care/info/#10d) | Adverse and unanticipated events. | N/A |
| **Discussion** |  |  |  |
|  | [#11a](https://www.goodreports.org/reporting-checklists/care/info/#11a) | Strengths and limitations in your approach to this case. | 4, 5 |
|  | [#11b](https://www.goodreports.org/reporting-checklists/care/info/#11b) | Discussion of the relevant medical literature. | 4, 5 |
|  | [#11c](https://www.goodreports.org/reporting-checklists/care/info/#11c) | The rationale for your conclusions. | 4, 5 |
|  | [#11d](https://www.goodreports.org/reporting-checklists/care/info/#11d) | The primary “take-away” lessons from this case report. | 5 |
| **Patient perspective** |  |  |  |
|  | [#12](https://www.goodreports.org/reporting-checklists/care/info/#12) | The patient can share their perspective on their case | N/A |
| **Informed consent** |  |  |  |
|  | [#13](https://www.goodreports.org/reporting-checklists/care/info/#13) | The patient should give informed consent. | 1 |

The CARE checklist is distributed under the terms of the Creative Commons Attribution License CC-BY-NC. This checklist was completed on 18. November 2022 using <https://www.goodreports.org/>, a tool made by the [EQUATOR Network](https://www.equator-network.org) in collaboration with [Penelope.ai](https://www.penelope.ai)
